# Achiral Molecular Recognition of Aromatic Position Isomers by Polysaccharide-Based CSPs in Relation to Chiral Recognition

**DOI:** 10.3390/molecules22010038

**Published:** 2016-12-28

**Authors:** Tohru Shibata, Satoshi Shinkura, Atsushi Ohnishi, Kazuyoshi Ueda

**Affiliations:** 1 Daicel Corporation, Life Science Development Center, Himeji 671-1283, Japan; sa_shinkura@jp.daicel.com (S.S.); at_ohnishi@jp.daicel.com (A.O.); 2 Graduate School of Engineering, Yokohama National University, Yokohama 240-8501, Japan; k-ueda@ynu.ac.jp

**Keywords:** chiral stationary phase, cellulose, amylose, position isomer, achiral, chromatography, simulation

## Abstract

Chromatographic separation of several sets of aromatic position isomers on three cellulose- and one amylose-based chiral stationary phases was performed to evaluate the potential of a polysaccharide-based chiral stationary phase (CSP) in the separation of isomeric or closely similar molecules, and to understand the interaction mechanism of this type of CSP with analytes. Their ability of molecular recognition was quite outstanding, but the selection rule was particular to each polysaccharide derivative. In the series of analytes, cellulose tris(4-methylbenzoate) and tris(3,5-dimethylphenylcarbamate) exhibited a contrasting selection rule, and the recognition mechanism was considered based on the computer-simulation of the former polymer.

## 1. Introduction

The urethanes and esters of polysaccharides, particularly of cellulose and of amylose, are well accepted as a class of the most potent chiral selectors [1,2,3]. We, in the course of attempts to separate a variety of hard-to-separate mixtures entrusted by customers, realized that chiral stationary phases (CSPs) very often exhibit nice recognition not only of chiral but also of structurally similar molecules. Such potential of CSPs, mostly for separating diastereoisomers, can be seen in scattered reports, some of which are referenced here [4,5,6,7,8,9,10,11,12,13,14,15,16,17,18]. If their achiral molecular recognition is something outstanding in comparison with that of commonly used achiral stationary phases, they may be utilized much more in achiral separation as well as chiral. This drove us to the systematic study of the power and the selection rule of achiral recognition to promote the application of these phases. Indeed, Regalado and Welch recently reported “the ‘trick’ of using CSPs in SFC (supercritical fluid chromatography) mode to separate challenging mixtures of closely related non-enantiomeric compounds” and mentioned that “many regular users of chiral SFC are surprised to learn of the power of this approach” [18]. Furthermore, we would like to add that it would make such utilization of these CSPs much easier if the characteristic feature of their molecular recognition would be better understood. 

There is another reason why we are interested in achiral molecular recognition. The mechanism of chiral recognition was elucidated for several combinations of a CSP and a chiral molecule [19,20,21]. A quantum mechanics or force-field calculation played an important role in predicting the interaction mechanism. However, we do not yet know what elementary interactions contribute to chiral recognition, other than hydrogen bonding. Even in a combination between an analyte and a CSP that cannot include hydrogen bonding, a fine chiral recognition often takes place (e.g., chiral separations of Trӧger’s base and methaqualone on cellulose tris(4-methylbenzoate)), which indicates the importance of some weaker interactions involved in the molecular recognition mechanism. To know these interactions should help us understand the general nature of a polysaccharide-based CSPs. We expected the retention behavior of a simple molecule which has a less conformational mobility and cannot form hydrogen bonds with the CSP would elucidate such weaker interactions.

Now we would like to report the separation of several sets of aromatic position isomers on four coating types of polysaccharide-based CSPs in this paper. More examples of isomer separation, including other types of isomers on coating and immobilized phases under HPLC and SFC conditions, are to appear on the website of Daicel [19].

## 2. Result and Discussion

### 2.1. Polysaccharide-Based CSPs

In this work, four prototypical polysaccharide-based CSPs, CHIRALCEL^®^ OB-H based on cellulose tribenzoate (hereafter abbreviated to “OB-H”), OJ-H on cellulose tris(4-methylbenzoate) (“OJ-H”), OD-H on cellulose tris(3,5-dimethylphenylcarbamate) (“OD-H”), and CHRALPAK^®^ AD-H based on amylose tris(3,5-dimethylphenylcarbamate) (“AD-H”) were chosen, though immobilized types are becoming prevalent in polysaccharide-based CSPs because of their serviceability. This is because “OB-H” and “OJ-H” often exhibit a characteristic retention behavior but are only available as coating type; the aim of this study is to understand the relation between the structure and selectivity of a polysaccharide-based CSP, rather than to establish practically useful chromatographic conditions. To know the general feature of polysaccharide-based CSPs, an octadecylsilyl phase (“ODS”) was also studied under reversed-phase conditions.

### 2.2. Separation of Some Sets of Aromatic Position Isomers

#### 2.2.1. Terphenyls and Triphenylene

The relative retention of terphenyl isomers and polycondensed aromatic hydrocarbons isomers are summarized in Table 1. While the separation of *o*-terphenyl (**1**) and triphenylene (**7**) is very often discussed as an index for shape recognition of a stationary phase, all of terphenyl isomers were studied as the analyte in this work. It can be seen that the separation factor for **1** and **7** cannot be the only measure of isomer recognition, as “OJ-H” exhibited the best recognition of three terphenyl isomers, while “OD-H” exhibited the largest separation factor (α) between **1** and **7**. Only “OB-H” exhibited the strongest retention of **2** among terphenyl isomers, and its preference of *meta*-substitution was also observed in dimethyl phthalate isomers. “AD-H” did only moderate recognition among isomers in spite of its excellent chiral recognition and “ODS” too.

#### 2.2.2. Polycondensed Aromatic Hydrocarbons (PAHs)

The retention of anthracene (**4**) and phenanthrene (**5**), and naphthacene (**6**) and triphenylene (**7**) were compared (Table 1). Here, the contrasting retention behaviors of “OD-H” and “OJ-H” become clearer. The former retained the nonlinearly condensed PAH more, and the latter retained the linearly condensed PAH between an isomeric pair, and the tendency is much more apparent in tetracyclic isomers, i.e., **6**, **7** than in tricyclic ones, i.e., **4**, **5**.

#### 2.2.3. Acetyl PAHs 

**Figure 1:**
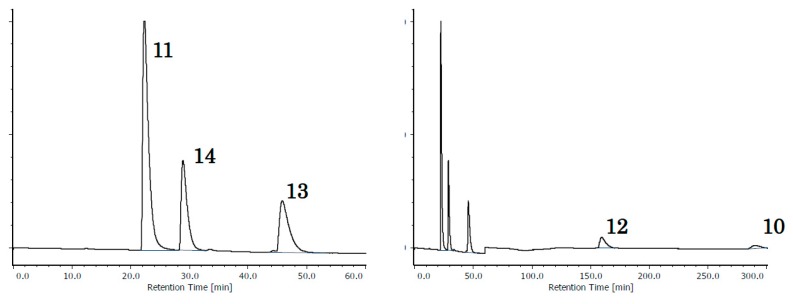

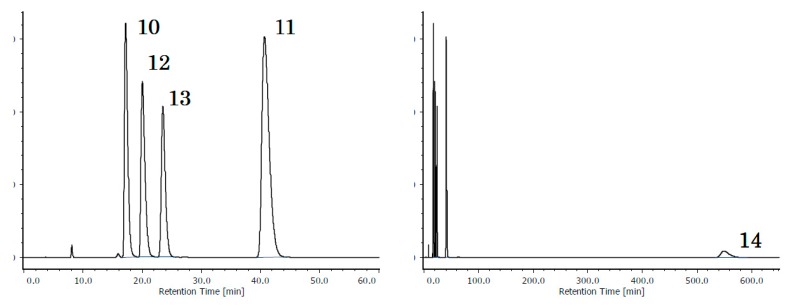
Chromatograms of isomeric acetyl phenanthrenes and anthracenes. Upper: On CHIRALCEL^®^ OJ-H. Lower: On CHIRALCEL^®^ OD-H. In each subfigure, the right side shows the total chromatogram and the left shows its 0–60 min range, enlarged. The elution order was totally inverse, except the one between **11** and **14**. The mobile phase was a mixture of hexane and 2-propanol in 100:1 (*v*/*v*) ratio. For other conditions, see Section 3. Materials and Methods.

The relative retention of acetylnaphthalenes, anthracenes, and phenanthrenes are summarized in Table 2, and the chromatograms of the five isomers of acetylanthracene (**10**, **11**) and acetylphenanthrene (**12**, **13**, **14**) on “OD-H” and “OJ-H” are given in Figure 1. Here, the selectivities of “OD-H” and “OJ-H” are opposite from each other, except for the order between **11** and **14**, where the nonplanarity of **11** may hinder retention to some extent. While it is not easy to quantitatively express the characteristics, it is clearly seen that “OD-H” tends to retain the two-dimensionally expanded isomer, and “OJ-H” tends to retain the linearly extended isomer. “AD-H” exhibited a moderate selectivity, but the selection rule is not clear and ODS exhibited only poor recognition.

### 2.3. Relation between Chiral and Achiral Recognition

**Scheme 2:**
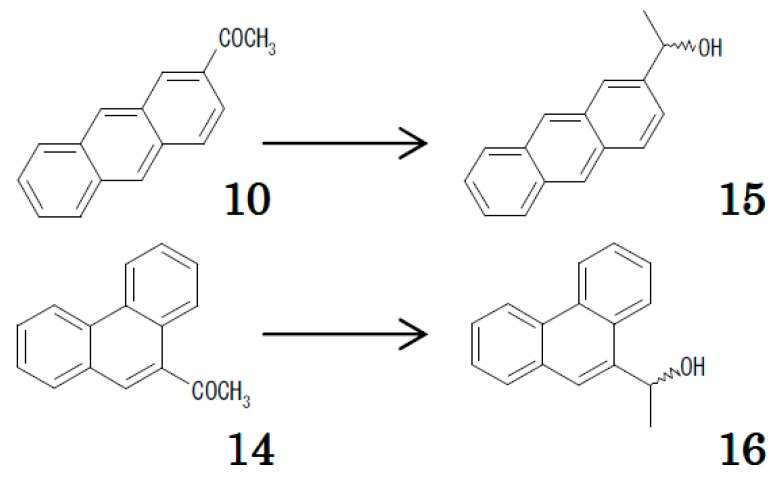
Ketone and the derived ethanol appearing in Table 3.

Here, our question was whether chiral recognition and achiral recognition are carried out independently or dependently. The suggestion arising from the contrasting selectivity of “OD-H” and “OJ-H” CSPs led us to study the chiral separation of the ethanol species **15** and **16**, respectively derived from the acetyl PAHs **10** and **14**, on the two CSPs (Table 3, Scheme 2). The ethanol derived from a ketone retained more on one of the CSPs was retained more on the CSP that is accompanied by a larger enantiomeric separation factor than the other CSP. It is rationalized that the frame of 2-substituted anthracene fits well to the “OJ-H” binding site, while 9-substituted phenanthrene fits into “OD-H”, and each coupling results in a better arrangement for chiral recognition. In other words, achiral and chiral recognitions occur in combination.

**Figure 2:**
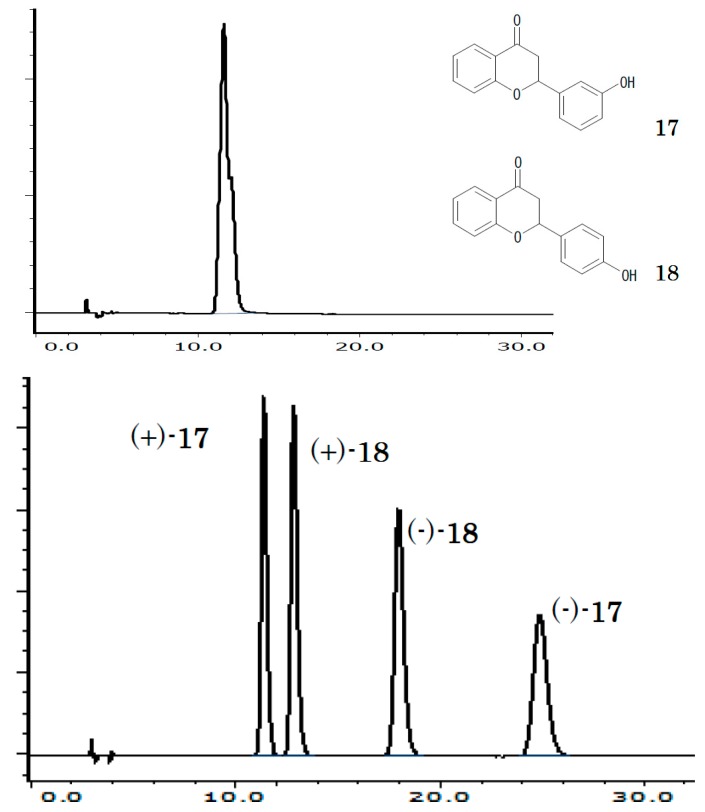
Chromatogram of racemic (±)-3'-hydroxyflavanone (**17**) and (±)-4'-hydroxy flavanone (**18**), upper on “OD-H” and lower on “AD-H”, each with hexane/ethanol/trifluoroacetic acid (80:20:0.1 *v*/*v*/*v*), 1.0 mL/min at 40 °C.

It often occurs that a successful chiral recognition is accompanied by diastereo-recognition or structural recognition, such as the example of flavanone isomers where “OD-H” recognizes neither but “AD-H” recognizes both (Figure 2). This suggests that structural and chiral recognitions are just an element of total molecular shape recognition.

### 2.4. Origin of the Selectivity

While some reports were published including computer-assisted simulation study of chiral recognition mechanism by polysaccharide-based CSPs [20,21,22], we have independently undertaken a simulation study to understand the remarkably different spectrum of separable racemates between “OB-H” and “OJ-H”. The study could also give a rationalization for the above mentioned achiral selectivity of “OJ-H”. Just a summary of the study is given hereafter, as the detail of the study has been submitted for publication [23].

#### 2.4.1. Conformer Contributing Molecular Recognition

**Figure 3:**
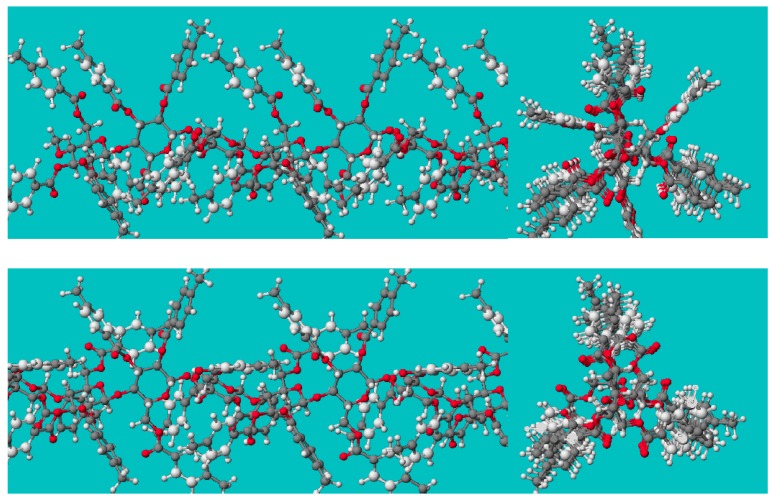
Two predicted conformers of cellulose tris(4-methylbenzoate) [23]. **Upper**: The bonds O(5)–C(6) and C(6)–O(6) taking *gauche* conformation, and C(5)–C(4) vs. C(6)–O(6) is *gauche* too (gg-conformer). **Lower**: The former taking *gauche* conformation and the latter taking *trans* conformations (*gt*-conformer).

The structure of “OB-H” and “OJ-H” polymers, cellulose tribenzoate (**CB**) and cellulose tris(4-methylbenzoate) (**CMB**), respectively, were optimized starting from the coordinates of **CB** reported by Zugenmaier [24], and two major energy minima were predicted for both derivatives (Figure 3). As is seen in the axial views, the 3-folded conformation of the cellulose backbone and the arrangement of 4-methylbenzoyloxy groups at glucosidic positions 2 and 3 are not much different, but the arrangement of the 4-methylbenzoyloxy group at glucosidic position 6 is quite different between the two conformers. In one conformer (Figure 3, upper, *gg*-conformer), the 4-methylbenzoyloxy group at this position is included in the line of those at positions 2 and 3, but in the other (Figure 3, lower, *gt*-conformer) it is out of the line, leaving a space in the line. In the perpendicular view to the molecular axis of the *gt* conformer, a space surrounded by glucopyranose rings and 4-methylbenzoyl groups can be seen. While in some reports concerning **CMB** [21,22] its *gt*-conformation was assumed, Zugenmaier had concluded that the *gg*-conformer is the deepest minimum of **CB**, and we obtained the same result for both **CB** and **CMB**. However, based on several considerations, we too predicted that the *gt*-conformer—which, we think, may be present at a certain population and/or under dynamic equilibria—must be the adsorption active form.

#### 2.4.2. Docking Simulation

**Figure 4:**
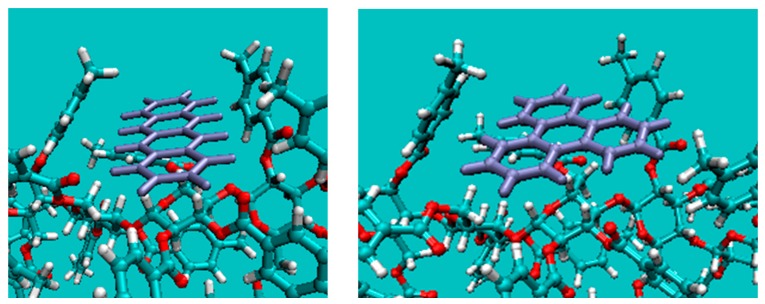
The predicted association of cellulose tris(4-methylbenzoate) respectively with naphthacene (**left**) and triphenylene (**right**).

The predicted binding site in Figure 3 (lower) looks like a gate, but from a different view angle (Figure 4), it has a long flat floor like a hallway. Then, docking simulation of **CB** and **CMB** with **6** and **7**, respectively, was performed to find energy minima by rote, and both analytes were predicted to bind the polymer at the aforementioned space. The binding energy of **CMB** was calculated to be −25.10 and −24.21 kcal, respectively with **6** and **7**, which agreed well with the chromatographic results where **6** is retained more. The total binding energy was divided into the destabilization of the polymer strained to accept an analyte and the binding energy between the strained polymer and the analyte. Thus, the much stronger binding of **6** than that of **7** with **CMB** could be rationalized mainly by the former (i.e., the larger deformation energy of the polymer to accept **7**). In other words, the intrinsic geometry of the **CMB** binding site is more suitable for accepting **6** than for accepting **7**. Besides, the poorer separation with the same elution order by **CB** was also traced by the simulation, and the better molecular recognition by **CMB** than by **CB** was attributed to the concert of 4-methyl hydrogen in binding (Figure 4).

**Figure 5:**
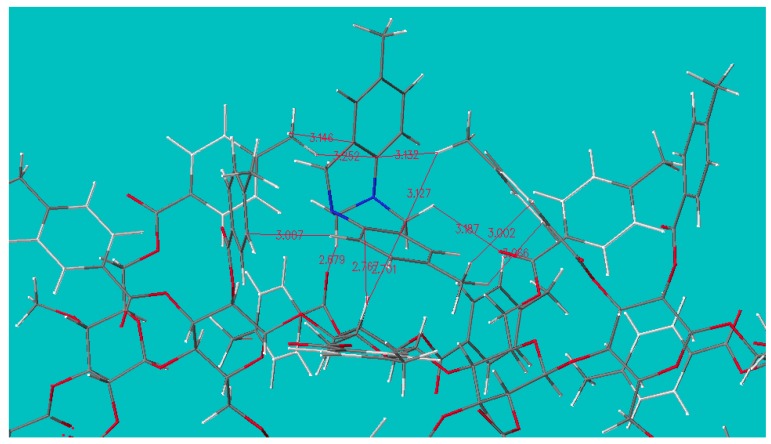
The predicted association of cellulose tris(4-methylbenzoate) and the more retained (*5R*, *11R*)-(−)-enantiomer of Trӧger’s Base. Atoms close together are connected by a thin line and the atomic distance is given. The close stacking between aromatic ring and glucose ring is notable. The contribution of benzoyl methyl groups can rationalize why “OJ” can separate Trӧger’s base enantiomer but “OB” cannot, and that of the methyl group of Trӧger’s base can explain why substituting the methyl group with either hydrogen or chlorine deteriorates retention and chiral recognition [26].

We have also studied the chiral separations of Trӧger’s base, mephobarbital, and methaqualone on “OJ-H”, and it was suggested that chiral recognition takes place in the aforementioned site of **CMB** (Figure 5). It is noteworthy that in each case of the simulation study, the α-face of a pyranose ring is predicted to stack with a cyclic π-system, which is a type of interaction attracting interest for its biological importance [25]. 

### 2.5. Difference between ODS Phase and Polysaccharide-Based CSPs

**Figure 6:**
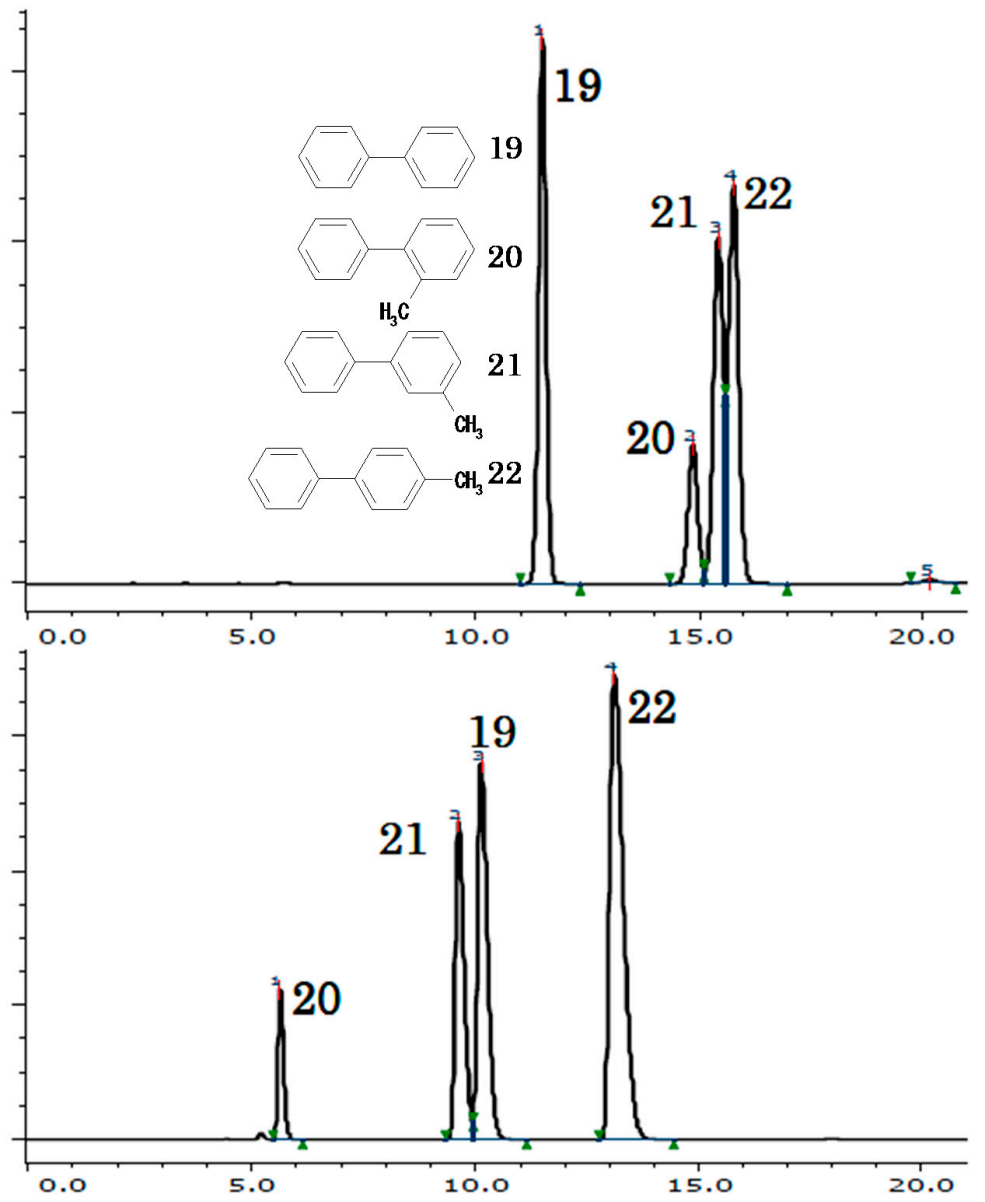
Chromatograms of biphenyl (**19**) and three methylbiphenyl isomers, 2-methylbiphenyl (**20**), 3-methylbiphenyl (**21**), and 4-methylbiphenyl (**22**). **Upper**: On L-Column^®^ with acetonitrile/water mixture (70:30 *v*/*v*). **Lower**: On CHIRALCEL^®^ OJ-H with hexane/2-propanol mixture (100:1 *v*/*v*), 1.0 mL/min.

Thus, having inferred the mechanism of molecular recognition, like inclusion, we then would like to consider the selection rule as the consequence, in comparison with the most accepted achiral phase, ODS. In the abovementioned comparisons between an ODS and a polysaccharide-based CSP, the latter usually exhibited a much superior isomeric recognition. However, this does not mean that the magnitudes of total molecular recognition of the CSP is larger than that of ODS but that the type of the molecular recognition by each is different. Figure 6 shows the chromatograms of biphenyl and methylbiphenyl isomers on “OJ-H” (under normal phase) and on “ODS” (under reversed phase). The elution pattern on “ODS” showed that retention increases with methylation regardless of the position of the methyl group, which is a reasonable result considering the hydrophobic property of a methyl group. In contrast, the elution pattern on “OJ-H” showed that the retention is strongly affected by the position of the methyl group rather than its presence or absence. Furthermore, the elution pattern on “OJ-H” was not essentially affected by a reversed-phase eluent (i.e., acetonitrile–water 70:30 *v*/*v* [22]), which is common in chiral recognition by a polysaccharide-based CSP [27]. Thus, each type of stationary phase exhibited an entirely different selection rule: one is physicochemical and the other is geometrical. Such an orthogonality must provide a useful coupling of stationary phases to attain a successful separation and/or a reliable confirmation of a purity.

## 3. Materials and Methods

### 3.1. Materials

Columns packed with polysaccharide-based chiral stationary phases are “CHIRALCEL OB-H”, “CHIRALCEL OJ-H”, “CHIRALCEL OD-H”, and “CHIRALPAK AD-H”, each 0.46 cm^id^ × 25 cm^L^, products of DAICEL Corporation (Osaka, Japan). An ODS column, “L-Column” (0.46 cm^id^ × 25 cm^L^), was purchased from Chemicals Evaluation and Research Institute of Japan (Bunkyo-ku, Tokyo, Japan). *o*-, *m*-, and *p*-terphenyl, anthracene, phenanthrene, triphenylene, 1- and 2-acetonaphthone, and 1-, 2-, and 3-methylbiphenyl were purchased from Tokyo Chemical Industry Co. (Chuo-ku, Tokyo, Japan); 2- and 9-acetylanthracene and 2-, 3-, and 9-acetyl phenanthrene were purchased from Sigma-Aldrich Corporation (St. Louis, MO, USA). Biphenyl and 2-propanol were purchased from Wako Pure Chemical Industries (Osaka, Japan) and hexane from Kanto Chemical Co. (Chuo-ku, Tokyo, Japan). Naphthacene was purchased from Nacalai Tesque Inc. (Kyoto, Japan). As naphthacene is usually contaminated with UV-active impurities, it was once chromatographed on CHIRALCEL OJ-H, and the major elution peak, the UV–vis spectrum of which coincides with available data, was collected and mixed with a triphenylene solution at an undetermined concentration and the 1 µL aliquot of the mixed solution was injected. 1-(2-anthryl)ethanol (**15**) and 1-(9-phenanthryl)ethanol (**16**) were derived from the corresponding acetyl PAH by sodium borohydride reduction in ethanol.

### 3.2. Instrumentation and Chromatographic Conditions

Chromatographic study on the CSPs was performed with a set of JASCO PU-980, AS-950, 865-CO, UV-975 instruments, products of JACSO Corporation (Hachioji, Japan). The mobile phase was a mixture of hexane and 2-propanol (100:1, *v*/*v*) unless otherwise noted, the flow rate was 1.0 mL/min, the column oven temperature was set at 25 °C, and the detection wavelength was 254 nm. A 1 μL aliquot of 0.1% analyte solution in the mobile phase was injected individually to roughly determine its elution time, and relative retention values were determined from the chromatogram of mixed analytes. When targeted peaks coalesced, data taken from individual analyses were adopted. 

### 3.3. Data Analysis

Relative retention (k’) and separation factor (α) were calculated with the equations below.

(1) k’ = (*V*/*V*_0_) − 1,

(2) α = k’_2_/k’_1_,

where V is the elution volume of an analyte and V_0_ is the column void volume. V_0_ was estimated from the injection shock caused by the injection of 5 μL aliquot of 2,2,4,-trimethylpentane and 2-propanol mixture (9:1 *v*/*v*), and the elution peak of tetrakis(trimethylsilyl)silane, both gave the value of 3.1 mL for every polysaccharide-based column. The subscript 1 and 2 in Equation (2) means the two analytes between which the separation factor is to be defined.

Chromatography on an ODS phase was performed under the same conditions to that with a polysaccharide-based column except the mobile phase, a mixture of acetonitrile and water (70:30, *v*/*v*). The V_0_ of the ODS column was estimated to be 2.29 based on the elution shock of acetonitrile and water (each 2 μL). While the method of determining V_0_ is a controversial issue, it is not of concern here.

The details of force-field calculation are to be published [23].

## 4. Conclusions

We studied the separation of several aromatic isomeric molecules on some polysaccharide-based CSPs, and the extremely wide range of relative retentions observed for isomers revealed their outstanding potential for isomeric recognition. We are convinced that they can be a powerful tool for difficult achiral separation, as some analytical chemists have pointed out [4,18]. 

The selection rule of the CSP is particular to each type. For example, “OD-H” and “OJ-H” often gave an inverse elution order in analyte sets studied here. In contrast to an ODS phase, which performs a recognition based on the physicochemical property of an analyte (i.e., hydrophobicity), a polysaccharide-based CSP exhibits a remarkable but rather unpredictable selectivity stemming from the geometrical fitting of the binding site of the selector and an analyte and, therefore, we would like to call it a geometrical recognition. Cyclodextrins are an established selector to perform such molecular recognition and the comparison of the selection rule between them and polysaccharide-based phases seems interesting.

The simulation study suggested the binding site of **CMB**, the “OJ-H” selector, to be a long space with a flat floor like a hallway consisting of 4-methylbenzoyl groups and glucose rings formed in the *gt*-conformer of the polymer. The simulation of chiral separation of Trӧger’s base and some other racemates by **CMB** revealed that a chiral recognition is also held in this site. Thus, chiral and achiral recognitions seem to be the different outcomes of the molecular geometry recognition held in this space. We expect the similar study on the “OD-H” polymer, which exhibits a selectivity contrasting to that of “OJ-H” polymer, **CMB**, will be very interesting and confirm the verity of the simulation.

The main interaction of **CMB** with a bare aromatic analyte is attributed to the accumulation of weak C–H···π interactions [23], and such a weak interactions may have more or less importance in a chromatographic separation on polysaccharide-based CSPs.

It must be emphasized that a CSP which looks incompetent for certain analyte series often exhibits a great potential for other series. It likely also stems from the particular shape and size of the binding site of the selector and understanding the feature of each site will make it easier to predict a suitable phase, not only for achiral, but also chiral recognition.

## Figures and Tables

**Scheme 1:**
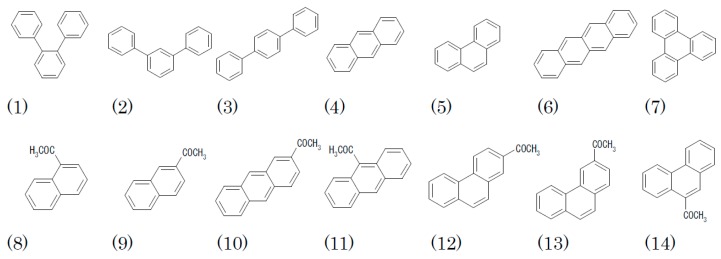
Structure of analytes appearing in Table 1 and Table 2.

**Table 1:** Relative retention of terphenyl and polycondensed aromatic hydrocarbon (PAH) isomers. See Scheme 1 for analyte structure.

CSP *	1	2	3	4	5	6	7
“OB-H”	0.35	3.52	1.35	1.18	1.16	7.00	2.01
“OJ-H”	0.51	1.76	4.23	6.96	5.29	52.6	3.99
“OD-H”	0.29	0.47	0.52	0.69	1.30	1.93	28.45
“AD-H”	0.18	0.51	0.58	0.43	0.41	2.08	0.86
“ODS”	8.42	10.26	11.45	6.79	6.14	15.72	11.30

* “CSP”: Chiral stationary phase; “OB-H”: CHIRALCEL^®^ OB-H; “OJ-H”: CHIRALCEL^®^ OJ-H; “OD-H”: CHIRALCEL^®^ OD-H; “AD-H”: CHIRALPAK^®^ AD-H; “ODS”: “L-Column”. See Section 3. Materials and methods for the column dimension, supplier, and analysis condition and see Section 2.1. Polysaccharide-based CSPs for the selector material.

**Table 2:** Relative retention of isomeric acetyl PAHs. See Scheme 1 for analyte structure.

CSP	8	9	10	11	12	13	14
“OB-H”	5.33	6.7	32.98	5.94	20.19	16.64	8.03
“OJ-H”	5.02	7.83	95.59	6.43	52.05	14.25	8.62
“OD-H”	3.67	3.30	4.79	12.78	5.77	6.86	186.26
“AD-H”	4.57	5.56	7.62	5.12	7.09	6.46	9.28
“ODS”	1.67	1.66	3.61	3.25	3.41	3.33	3.24

**Table 3:** Relative retention (k’) of 2-acetylanthracene (**10**), 9-acetylphenanthrene (**14**), and the racemic alcohols **15** and **16** derived from each.

CSP	Analyte			
	10	15 Enantiomers	14	16 Enantiomers
“OJ-H”	96	32.4, 95.2 (α = 2.94)	8.6	4.35, 4.68 (α = 1.07)
“OD-H”	4.8	2.42, 4.87 (α = 2.01)	186	20.8, 123 (α = 5.88)

**10** and **14** were chromatographed with hexane/2-propanol (100:1 *v*/*v*) mixture, and **15** and **16** with hexane/2-propanol (9:1 *v*/*v*) mixture. Every k’ was calculated with the V_0_ value of 3.1 mL.
